# Comparing Deceased Organ Donation Performance in Two Countries that Use Different Metrics: Comparing Apples With Apples

**DOI:** 10.3389/ti.2022.10461

**Published:** 2022-05-13

**Authors:** Luke Milross, Chloe Brown, Laura Gladkis, Kylie Downes, Melissa Goodwin, Susanna Madden, Mark McDonald, Lucinda Barry, Helen Opdam, Alex Manara, Dale Gardiner

**Affiliations:** ^1^ Organ and Tissue Donation and Transplantation, NHS Blood and Transplant, Bristol, United Kingdom; ^2^ Organ and Tissue Authority, Canberra, NSW, Australia

**Keywords:** transplantation, organ donation, performance, auditing, reporting, metrics, definitions

## Abstract

Organ donation networks audit and report on national or regional organ donation performance, however there are inconsistencies in the metrics and definitions used, rendering comparisons difficult or inappropriate. This is despite multiple attempts exploring the possibility for convergently evolving audits so that collectives of donation networks might transparently share data and practice and then target system interventions. This paper represents a collaboration between the United Kingdom and Australian organ donation organisations which aimed to understand the intricacies of our respective auditing systems, compare the metrics and definitions they employ and ultimately assess their level of comparability. This point of view outlines the historical context underlying the development of the auditing tools, demonstrates their differences to the Critical Pathway proposed as a common tool a decade ago and presents a side-by-side comparison of donation definitions, metrics and data for the 2019 calendar year. There were significant differences in donation definition terminology, metrics and overall structure of the audits. Fitting the audits to a tiered scaffold allowed for reasonable comparisons however this required substantial effort and understanding of nuance. Direct comparison of international and inter-regional donation performance is challenging and would benefit from consistent auditing processes across organisations.

## Introduction

Organ transplantation is a lifesaving, life-transforming intervention which often is the only effective treatment available to patients with end-stage organ failure. Such patients rely on a limited supply of organs and experience high mortality and significant morbidity whilst waitlisted (1). Supply is influenced both by the size of the potential donor pool and critically the efficacy of its conversion into actual donors (2). Conversion broadly depends on healthcare system resources and cultural factors and is facilitated through donor identification, referral and approach, community attitudes to donation, donor physiological support and transplant unit acceptance practices. Countries with advanced donation systems have organ donation organisations which lead in the assessment of national/regional donation conversion performance, collecting data to identify barriers to donation, direct interventions and measure the effect of their implementation.

Meaningful comparison of national/regional donation metrics might allow for sharing of best practice and overall improvement of donation performance. Countries with low conversion rates could learn from practices of countries with better performance (3). However, difficulties exist in comparisons due to inconsistencies in the definitions and metrics used as performance indicators (4). Indeed, a recent US study showed significant variability in the performance rankings of organ procurement agencies depending on which donation metrics were used (5).

The “Critical Pathway for Deceased Donation,” the outcome of a multi-national initiative held between 2008–10, was aimed to provide a solution to this issue by providing a set of common definitions to guide consistency in reporting of donation performance (6). However, while the Critical Pathway was welcomed, the goal of common international definitions has not been realised and many nations have witnessed divergent evolution in the audit of donation performance. We aimed to explore this issue through a collaboration between the national donation organisations of the United Kingdom and Australia, both countries which contributed to the development of the critical pathway. In this point of view, we will outline the critical pathway for deceased donation, the history of the development of our individual auditing tools and finally, investigate the degree of comparability between our donation definitions and metrics.

## The Critical Pathway for Deceased Donation

The critical pathway for deceased donation was developed by a multi-national collective at the Madrid Resolution on Organ Donation and Transplantation (7) and published by Dominguez et al. in 2011 (6). It outlines a series of definitions which enable all “possible deceased organ donors” to be quantified, including definitions for “potential” donors, “actual” donors and “utilised” donors. A similar template was recently suggested for European tissue donation (8). The value of this structured approach to donation networks is its ability to pinpoint where unrealised donation opportunities occur along the pathway. Where cases of avoidable unrealised donation are identified, interventions can be targeted to increase rates of donation.

Inclusion in the “possible deceased organ donor” pool is defined by the critical pathway as “A patient with devastating brain injury or lesion or a patient with circulatory failure and apparently medically suitable for organ donation”(6). The pathway then splits into two components, separating into donation after brain death (DBD) and donation after circulatory death (DCD) pathways. There are four major steps to each pathway (Table 1); “Potential,” “Eligible,” “Actual” and “Utilised” DBD/DCD donors.

**TABLE 1:** Critical pathway for deceased donation definitions—adapted from Dominguez et al. (2011)^6^.

Common term	DBD component	DCD component
Potential	Potential DBD donor: A person whose clinical condition is suspected to fulfil brain death criteria	Potential DCD donor:
		A. A person whose circulator and respiratory functions have ceased and resuscitative measures are not to be attempted or continued, or
		B. A person in whom the cessation of circulatory and respiratory functions is anticipated to occur within a time frame that will enable organ recovery
Eligible	Eligible DBD donor: A medically suitable person who has been declared dead based on neurological criteria as stipulated by the law of the relevant jurisdiction	Eligible DCD donor: A medically suitable person who has been declared dead based on the irreversible absence of circulatory and respiratory functions as stipulated by the law of the relevant jurisdiction, within a time frame that enables organ recovery
Actual	Actual DBD donor: A consented eligible donor:	Actual DCD donor: A consented eligible donor:
	A. In whom an operative incision was made with the intent of organ recovery for the purpose of transplantation, or	A. In whom an operative incision was made with the intent of organ recovery for the purpose of transplantation, or
	B. From whom at least one organ was recovered for the purpose of transplantation	B. From whom at least one organ was recovered for the purpose of transplantation
Utilised	Utilised DBD donor: An actual donor from whom at least one organ was transplanted	Utilised DCD donor: An actual donor from whom at least one organ was transplanted

## The Development of the UK and Australian Donation Audits

The development of the potential donor audit (PDA) in the UK followed the publication of a study auditing DBD potential in intensive care units (ICUs) which estimated a possible 20% increase in deceased kidney donation based on prompt testing for brain stem death (9). Following this publication, the first UK PDA, auditing the DBD pathway, was established in 2003. Since then, the PDA inclusion criteria have been extended, firstly in 2009 to also audit the potential for DCD donation and include deaths in emergency departments (EDs), and next in 2013 when the age criteria were extended from 75 years and under to 80 years and under. Enhancements to the PDA were made in 2020 to capture more informative data on the medical suitability of eligible DCD donors and further detail on the donation decision conversations. Since this time, data are collected via an app and can be entered in real time. Data are input and validated by Specialist Nurses in Organ Donation (SNODs), employed by NHS Blood and Transplant (NHSBT), who are embedded in the individual hospitals.

Early audits of hospital deaths occurred in several states in Australia with the aim of quantifying the potential for organ donation, focusing on identifying missed donor cases (10-12). Most missed opportunities for donation occurred in severely brain injured patients who, due to poor prognoses, had treatment withdrawn in the ED or ICU. The first national audit occurred during a National Organ Donation Collaborative from 2006–09. In 2009, a national reform began that included the establishment of a national agency, the Organ and Tissue Authority and the state-based DonateLife Network. The DonateLife Audit was developed as a monitoring tool with retrospective review of all hospital patient deaths with donor potential. A new web-based tool was implemented in 2012 that included fields for donor physiology and organ function, providing more detailed information about donor organ suitability for transplantation. The audit provides a means of optimising clinical practice both at a local and national level, identifying cases with learning points for local case review and providing national, jurisdictional and hospital level data on measures such as the donor pool, and rates of consent and donation (13). Regular internal reporting enables monitoring of clinical practice improvement including the routine referral to donation services of patients at medical consensus of end-of-life and utilisation of a best practice approach to offering donation to families (14). The audit is completed by donation specialist staff and is undertaken in most Australian hospitals with donor potential.

## A Comparison of UK and Australian Definitions and Metrics Used in Donation Reporting

Over 2020–2021, we conducted a series of virtual meetings aiming to compare national methods, definitions and metrics used for data collection and reporting of national deceased donation performance. Tables were created outlining the definitions used in DBD and DCD pathways set out by the “Critical Pathway for Deceased Donation” (6) in the first column, with further columns left blank for population by nearest equivalent definitions from Australian and UK official reference documents. These included the “Potential Donor Audit Report 2019–20” from NHS Blood and Transplant, UK and the “DonateLife Audit Standard Operation Procedure” used by the Organ and Tissue Authority in Australia. Side-by-side definitions allowed for in-depth discussion within the group surrounding similarities and differences between definitions used. Minutes were taken and differences and similarities synthesised through discussion across subsequent meetings.

General differences between the auditing structures were immediately apparent (Table 2). Estimating the potential donor pool is essential to any donation audit and the first challenge is that the two national audits cast differently sized nets in the denominator of audited deaths. In the UK, deaths are only audited if they physically occurred within the ICU or ED. In Australia, this is extended to deaths due to irrecoverable brain injury occurring anywhere in hospital within 24 h of being in an ICU or ED. The audits also differ slightly in age at death range captured. Both audits capture deaths from 28 days to 80 years, however the Australian audit also includes patients who were referred for consideration of organ donation outside these criteria, for example those above 80 years old where a family request was made and where donation was considered feasible by attending staff. Differing inclusion criteria mean that when it comes to comparing the possible donor pools between countries, we could only proceed by restricting inclusion to death in ICU alone.

**TABLE 2:** Differences in audited deaths included in the UK and Australian donation audits.

	United Kingdom	Australia
Inclusion criteria	Deaths under 80 years old occurring in intensive care OR emergency department (excluding deaths in neonatal ICU)	Deaths under 80 years old or >28 days old occurring in intensive care or emergency departments OR occurring anywhere in hospital within 24 h of presence in intensive care OR emergency department where irrecoverable brain injury present. Additional inclusion of patients >80 yr if formal request for consideration of donation placed by family and donation considered feasible by attending staff
Data pathway structure	DBD and DCD data audited separately	DBD and DCD data combined in audit
Network Organisation	National, centralised service: “Statistics and Clinical Research department, NHS Blood and Transplant”	National, centralised service: the “Organ and Tissue Authority” (OTA) which maintains a web-based auditing tool capturing approx. 98% of deceased donation activity in Australia
Data Collection and input	Specialist Nurses in Organ Donation embedded in individual hospitals	Nurse donation specialists embedded in individual hospitals or through outreach roles in smaller hospitals without permanent embedded staff

**FIGURE 1:**
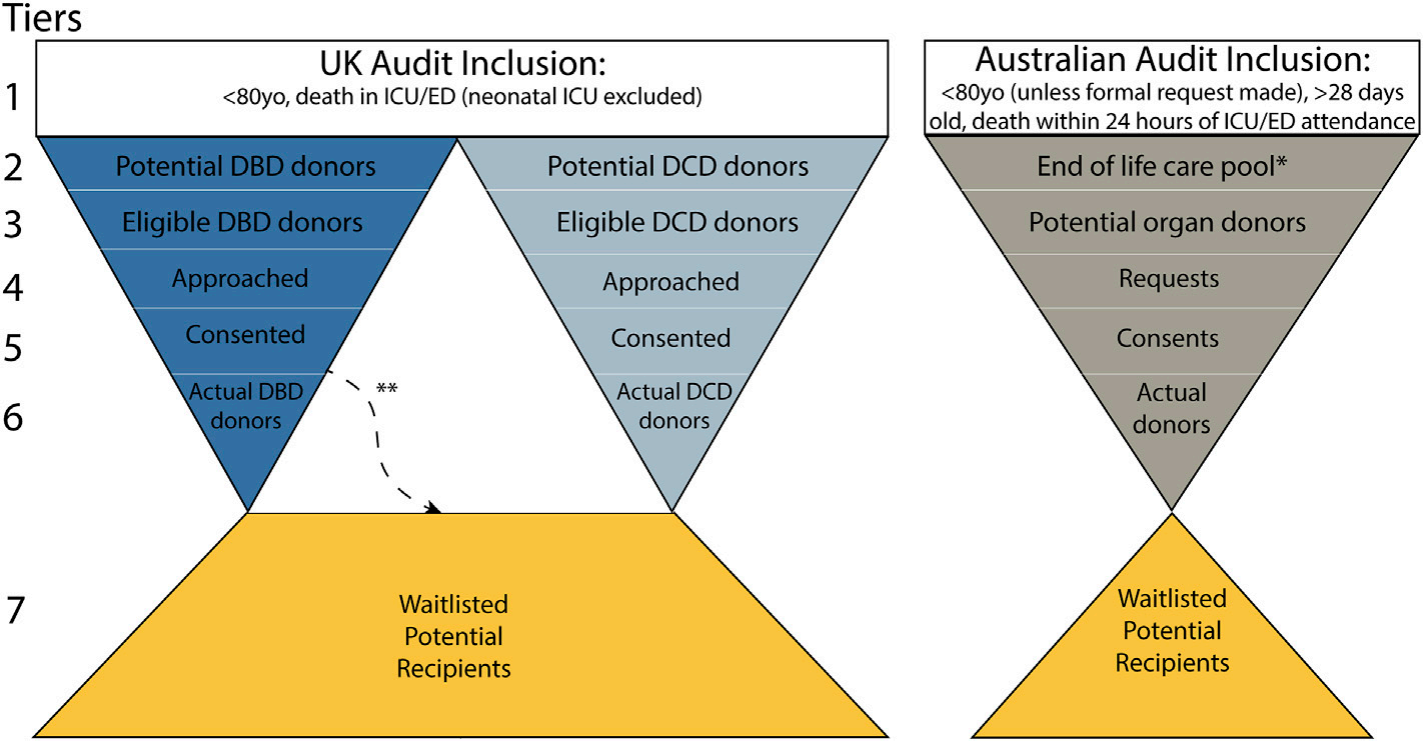
Structure of donation performance audits in the UK (left) and Australia (right). *Not publicly available, **refers to “Actual donors: DCD,” a small subset of those who are brain dead who enter a DCD pathway by specific request of family.

The basic structure of the audit also differed. In the UK, when DBD and DCD cases are audited they feed into separate streams of data collection (similar to the Critical Pathway) whereas in Australia these streams are combined (Figure 1).

Despite some differences in terminology used between countries, both audits could be fitted to seven major tiers (Figure 1). The general inclusion criteria (Tier 1) already represented an uneven starting point for comparisons, and differences continued throughout the tiers. Table 3 outlines specific differences in the UK and Australian donation audits in Tiers 2–6. Tier 2 represents the first group in each audit which is deemed to have donation potential, thus warranting inclusion for further evaluation. In the UK, potential DBD and DCD donors are separate and feed down the audit as such whereas in Australia these groups are combined into an “End-of-Life Care Pool.” The Australian end-of-life care pool contains patients confirmed brain dead (or likely to have fulfilled criteria for brain death), or had treatment withdrawn and where death was anticipated, thus combining the DBD and DCD streams.

**TABLE 3:** Specific differences in the UK and Australian donation audits.

Tier	UK—DBD	UK—DCD	Australia	Comments
2	“Potential DBD donor”	“Potential DCD donor”	“End-of-life care pool”	-Differing terms
	A patient who meets all four criteria (coma, ventilated, fixed pupils, apnoeic) for neurological death testing excluding those not tested due to reasons “cardiac arrest despite resuscitation,” “brainstem reflexes returned,” “neonates—less than 2 months post term”	A patient who had treatment withdrawn and death was anticipated within 4 hours	Any patient who meets the following criteria:-Confirmed or suspected brain death-Withdrawal of one or more of mechanical ventilation, artificial airway, mechanical circulatory support prior to death as part of the process of end-of-life care-A decision was made regarding organ donation	-DBD: Australian audit combines suspected brain dead and those confirmed via testingDCD: UK places time restriction of anticipated to 4 hours-“End-of-Life Care Pool” data not publicly available
3	“Eligible DBD donor.” Patients for whom death was confirmed following neurological tests and who had no absolute medical contraindications to solid organ donation	“Eligible DCD donor”Patients who had treatment withdrawn and death was anticipated within 4 hours, with no absolute medical contraindications to solid organ donation	“Potential donor”Any of the “End-of-Life Care pool” who were medically suitable/had no absolute medical contraindications to solid organ donation	-Differing terms-Neurological tests to confirm brain death for inclusion in category in both countries-Inclusion subject to differences in lists of absolute medical contraindications/medical suitability
4	“Approached DBD donors.” Eligible DBD families approached for consent/authorisation for donation	“Approached eligible DCD donors.” Eligible DCD donor families approached for consent/authorisation for donation	“Requests”Count of all cases where organ donation was discussed with the family and a final decision of consent or decline was made. Includes all requests, regardless of age or potential donor status, except cases where family was advised of lack of donor suitability	-Differing terms-Differing denominators with UK using eligible DBD/DCD donors only-UK also uses both terms “consent” and “authorisation” owing to different legislation in Scotland
5	“Consented DBD donors.” Families or nominated/appointed representatives of eligible DBD donors approached for formal organ donation discussion where consent/authorisation was ascertained	“Consented eligible DCD donors.” Families or nominated/appointed representatives of eligible DCD donors approached for formal organ donation discussion where consent/authorisation was ascertained	“Consents”Consent for organ donation is given by the family or next of kin. Cases where the family is advised of lack of donor suitability are not included	-Congruent in inclusion of actual family donation conversations in cases which had no absolute or prior identified medical contraindications
6	“Actual donors: DBD”: Consented, eligible DBD pathway patients who became actual DBD donors as defined by organ retrieval with the intention to transplant (unless returned to donor where considered unsuitable)	“Actual DCD donors”:Consented, eligible DCD pathway patients who became actual DCD donors as defined by organ retrieval with the intention to transplant (unless returned to donor where considered unsuitable)	“Actual donors”:A person for whom the organ retrieval procedure commenced in the operating room (with surgical incision) for the purpose of transplantation. This includes donors who may have been deemed medically unsuitable during surgery or after the removal of organs	-Actual donation defined at “knife to skin” of donor in Australia and “organ retrieval with the intention to transplant” in UK.-Select few in DBD pathway in UK who became DCD donors due to specific requests of family reported in audit. This does occur in Australia however is not publicly reported

There were differences in the inclusion criteria of potential DBD- and DCD-pathway patients. For DBD in the UK, Tier 2 contains those suspected of brain death and meet criteria for formal neurological death testing whereas in Australia Tier 2 captures both suspected and confirmed brain dead patients. For DCD in the UK, a timeframe is applied to the potential DCD donor definition with inclusion if death was anticipated within 4 hours of withdrawal of life-sustaining treatment whereas Australia includes deaths which actually occurred within 6 h of withdrawal (or longer if DCD was planned but death did not occur within 6 h).

Tier 3 represents those in Tier 2 who are then deemed medically suitable with no absolute contraindications to donation. The UK refers to these patients as “Eligible DBD/DCD donors” as per the Critical Pathway (6) whereas Australia uses the term “Potential donors.” For inclusion of those in the brain death pathway in Tier 3, confirmation of brain death by formal neurological testing is essential to both audits. Data is impacted at Tier 3 due to differences in exclusion criteria outlined by nationally accepted lists of absolute contraindications.

Tier 4 refers to the interaction between donor families and healthcare staff including donation coordinators, nurses and hospital doctors. In the UK, donation coordinators are referred to generically as Specialist Nurse-Organ Donation (SNOD) and in Australia the term Donation Specialist Nurse encompasses a number of slightly varying roles. At this tier, differing semantics are used, however both “Approach” (UK) and “Request” (Australia) are used in the audit which refers to family approaches to offer donation. Where these definitions do differ is in their denominator, with only those deemed eligible included in the UK whereas in Australia it is all discussions held, including those which may have been raised by families or led by ICU staff where donation was initially considered feasible although ultimately the person was not suitable.

Tier 5 is the consent rate of those families approached or requested for donation. The combined DBD/DCD Australian figure means comparison of specific consent, between the two types of deceased organ donation, cannot be readily achieved such as in the UK.

Tier 6 counts where donation is considered to have taken place. In the UK, “actual donor” status is defined by organ retrieval with the intention to transplant whereas in Australia cases are included at the point of “knife to skin” of the donor, both irrespective of actual utilisation (implantation) of organs. A final difference in audit structure occurs here as the UK reports on the small proportion of those included in the DBD pathway who actually proceed down a DCD pathway due to specific requests from the family to be present when the heart stops beating. Such cases also occur in Australia in practice.

## Comparison of Real Data—What Can Be Reasonably Compared?

We next examined real data collected by both national audits (Table 4). The 2019 calendar year was chosen as this was the most recent year where donation activity was not impacted by the COVID-19 pandemic. To proceed, the DBD and DCD streams in the UK audit needed to be totalled for equivalence to the corresponding Australian tiers. We were able to compare figures for the possible donor pool (Tier 1) by adjusting the catchment to include only deaths occurring within ICUs. However, this by necessity, excluded deaths associated with other locations such as EDs and wards and thus underestimates the true donor pool (11). Where appropriate, data was provided in absolute numbers as well as in per million population (pmp) however we note population age distribution impacts national donation potential (15). This figure is also impacted by proportion of donation-compatible deaths, for example differing due to variable cerebrovascular disease and traffic accident mortality (16).

**TABLE 4:** Comparison of 2019 donation activity data in the UK and Australia across tiers. Population estimate used for per million population (pmp) calculations were 66.8 million in the UK and 25.37 in Australia for 2019.

Tier	Corresponding metric	UK (DBD + DCD)	Australia
1	Deaths in chosen location (ICU)	22688 (339 pmp)	5990 (234 pmp)
2	Potential donors (UK) or EOL care pool (Aus)	Not included	Not included (not publicly available)
3	“Eligible” (UK)/“Potential” (Aus)	5844 (87 pmp)	1309 (51 pmp)
4	“Approached” (UK)/“Requested” (Aus)	3351 (50 pmp)	1224 (48 pmp)
5	Consents	2276 (34 pmp)	756 (30 pmp)
	Consent rate	67.9%	62%
6	Actual donors	1624 (24 pmp)	548 (22 pmp)

## Discussion

Direct comparison of UK and Australian deceased organ donation data was challenging due to differences in the metrics and definitions used by the national donation networks. A tiered structure allowed approximations at each step of the pathway and subsequently, certain comparisons could be cautiously made. Interpretation of comparisons requires detailed understanding of the way data is derived, collection methods, flow and the relationships between data points.

Difficulties in comparing national donation performance is not a new issue. Jansen et al. (2009) found significant heterogeneity in definitions used for “potential organ donor” and “refusal rate” across 11 European countries (4). They concluded non-uniform definitions meant that comparisons were not appropriate and called for shared definitions. In the United States, non-standardised, inconsistent, self-reported metrics reported by Organ Procurement Organisations (OPOs) also make interregional performance assessments problematic (5,17,18). As pointed out by Goldberg *et al.* (2019) this is an issue of fairness as these metrics inform interventions which could improve access in truly underperforming states. Canada also has difficulties with a lack of standardisation possibly due to its provincially-administered healthcare system (19).

Many initiatives have attempted to establish and promulgate a set of standard definitions and metrics which measure donation performance. Most notably, the multi-national collaborative led by Dominguez et al. (2011) established the “critical pathway for deceased donation” which played an important role in providing a universal framework for the process of deceased organ donation (6). However, donation practices constantly evolve, necessitating continuous reassessment of benchmarking practices. A recent ‘call to action’ from the European Kidney Health Alliance argued there is work to be done and recommended establishing appropriate comparative tools (3).

Our group attempted to take up the mantle of this work. From our minutes, “The goal is the concept of potentially using our two databases and trying to bring them together so that we can actually have comparative metrics.” It was noted that the two audits, “…have probably evolved in different directions.” When comparing our audits, we first noted there were several significant general differences in their structure. The starting points varied due to differing inclusion criteria in estimating the “possible” donor pool. We also note that not all ICUs and EDs report all deaths where organ donation is possible in a consistent and standardised way. To identify the full depth of this pool would require an audit of all hospital deaths nationally (11). For the purposes of our review, we approximated our data by only considering deaths in ICU though this is inconsistent with our actual practice and underestimates the donor pool. Our second major difference was that when DBD and DCD cases are audited they feed into separate streams of data in the UK whereas in Australia they are reported in a combined fashion. A strength of separate reporting is the ease in external assessment of DCD implementation. DCD has been shown as a way to increase donation activity and contributes substantially to overall donation numbers (20) and therefore may benefit from separate monitoring. However, a weakness in stream separation lies in accounting for the small number of potential donors where the donation process was stopped prior to the point where the pathway was completely differentiated or, in the data collection phase, where it was not possible to allocate them retrospectively to a pathway.

We developed a tiered system based on the critical pathway for deceased donation to compare the definitions and metrics used by our audits. At almost every tier there were different uses of terminology and nuance in metrics. It was felt that much of the differences found were in the way data was reported rather than collected and that internal data could be produced which would more readily match the counterpart organisation’s data. Undertaking this work itself did help with interpreting each counterpart’s figures and some comparisons were felt to represent reasonable approximations.

There are several limitations with auditing donation performance in general. The audits attempt to simplify the messy real world of variably unfolding patient scenarios and different clinician practices and record-keeping. Difficulties arise in capturing scenarios outside of the expected ‘order of events’, for example where families are approached at earlier stages such as prior to brain death testing. Furthermore, the audits variably combine elements of retrospective data collection as well as data collection which is actively and purposefully collected during the donation process. For example, when recording potential DCD donors, the UK approach would be to include “A patient who had treatment withdrawn and death was anticipated within 4 hours”, this relying on the clear recording of “anticipation” of death during the donation process for later retrospective data collection. In other words, this element of the audit is conducted prospectively but collected retrospectively. In Australia, the observation that death occurred within 6 h of withdrawal of cardio-respiratory support (or beyond 6 h if donation had been planned) is the trigger for inclusion which necessitates the retrospective approach.

We also discussed the mutual development of “quality metrics”, including tracking characteristics of the donation conversation, from formalised pre-discussion planning sessions to presence of donation specialise staff. Notably, donation coordinator nursing staff involvement in donation conversations is implicated in increasing DBD and DCD consent rates (21).

Clearly, moving towards a shared reality, “international language” and uniform metrics is desirable. Table 5 outlines our suggestions for the immediate steps and future directions which can be taken which include further work between our organisations and others. In the future, international donation networks could audit a standardised pool of potential donors, capturing all deaths using a global coding system integrating digital time stamps and in a digitalised, user-friendly system. Metrics could then be generated from shared definitions and reported in multiple formats including absolute numbers, adjustments made for per million population and even considerations for adjustments made for population age distribution and “mortality profiles” (16).

**TABLE 5:** Immediate actions and future directions.

• The most meaningful comparisons between the UK and Australian donation organisations begin at “Tier 4,” or the number “approached” or “requested” for donation. Further collaborations between our organisations should focus on downstream data comparisons including consent and conversion rates
• Invite and encourage dialogue between other organ donation organisations interested in updating or evolving their audits by establishing a working group which would routinely meet at a recurring international conference such as the International Society for Organ Donation and Procurement (ISODP) Congress
• The use of standardised definitions and metrics by databases which collect and publish data on organ donation and transplantation activity such as the Global Observatory on Donation and Transplantation (GODT)
• Encourage the use of side-by-side descriptive information alongside data points in publications which aid the reader in understanding how each data point was derived

We found that comparison of deceased organ donation data between two countries, which at first glance have similar culture and donation practice, was extremely challenging due to differences in our metrics and definitions. This would be compounded when comparing with even more countries and organ donation organisations. However, this work is essential if we are to search widely for solutions and learn from our partners when addressing the shortage of organs for transplantation. We do know that our goal is the same: the minimisation of unrealised potential donors. We therefore encourage, invite and hope to foster larger collaborative efforts from this international audience towards the goal of convergent evolution of definitions and metrics. This work will become increasingly relevant as practices in organ donation and transplantation evolve with society and time. It’s time to compare apples with apples when reporting donation performance.

## Data Availability

The original contributions presented in the study are included in the article/Supplementary Material, further inquiries can be directed to the corresponding author.

## References

[1] Lewis A Koukoura A Tsianos G-I Gargavanis AA Nielsen AA Vassiliadis E. Organ Donation in the US and Europe: The Supply vs Demand Imbalance. Transplant Rev (2021) 35(2):100585. 10.1016/j.trre.2020.100585 33071161

[2] Matesanz R. Factors that Influence the Development of an Organ Donation Program. Transplant Proc (2004) 36(3):739–41. 10.1016/j.transproceed.2004.03.025 15110647

[3] Vanholder R Domínguez-Gil B Busic M Cortez-Pinto H Craig JC Jager KJ Organ Donation and Transplantation: a Multi-Stakeholder Call to Action. Nat Rev Nephrol (2021) 17(8):554–68. 10.1038/s41581-021-00425-3 33953367 PMC8097678

[4] Jansen NE Haase-Kromwijk BJJM van Leiden HA Weimar W Hoitsma AJ. A Plea for Uniform European Definitions for Organ Donor Potential and Family Refusal Rates. Transpl Int (2009) 22(11):1064–72. 10.1111/j.1432-2277.2009.00930.x 19686462

[5] DeRoos LJ Zhou Y Marrero WJ Tapper EB Sonnenday CJ Lavieri MS Assessment of National Organ Donation Rates and Organ Procurement Organization Metrics. JAMA Surg (2021) 156(2):173–80. 10.1001/jamasurg.2020.5395 33263743 PMC7711570

[6] Domínguez-Gil B Delmonico FL Shaheen FAM Matesanz R O’Connor K Minina M The Critical Pathway for Deceased Donation: Reportable Uniformity in the Approach to Deceased Donation. Transpl Int (2011) 24(4):373–8. 10.1111/j.1432-2277.2011.01243.x 21392129

[7] Tharu R. The Madrid Resolution on Organ Donation and Transplantation. Transplantation. 2011;91:S29–31. 10.1097/01.tp.0000399131.74618.a5 21633281

[8] Sandiumenge A Domínguez‐Gil B Pont T Sánchez Ibáñez J Chandrasekar A Bokhorst A Critical Pathway for Deceased Tissue Donation: a Novel Adaptative European Systematic Approach. Transpl Int (2021) 34(5):865–71. 10.1111/tri.13841 33559299 PMC8251811

[9] Gore SM Cable DJ Holland AJ. Organ Donation from Intensive Care Units in England and Wales: Two Year Confidential Audit of Deaths in Intensive Care. Br Med J (1992) 304(6823):349–55. 10.1136/bmj.304.6823.349 1540732 PMC1881246

[10] Hibberd AD Pearson IY McCosker CJ Chapman JR Macdonald GJ Thompson JF Potential for Cadaveric Organ Retrieval in New South Wales. Br Med J (1992) 304(6838):1339–43. 10.1136/bmj.304.6838.1339 1611330 PMC1882046

[11] Opdam HI Silvester W. Identifying the Potential Organ Donor: an Audit of Hospital Deaths. Intensive Care Med (2004) 30(7):1390–7. 10.1007/s00134-004-2185-9 15024567

[12] Opdam HI Silvester W. Potential for Organ Donation in Victoria: an Audit of Hospital Deaths. Med J Aust (2006) 185(5):250–4. 10.5694/j.1326-5377.2006.tb00554.x 16948619

[13] Organ and Tissue Authority. Canberra, ACT: 2020 Australian Donation and Transplantation Activity Report (2020)). Available at: https://www.donatelife.gov.au/sites/default/files/2020_australian_donation_and_transplantation_activity_report.pdf (Accessed 1 24, 2022).

[14] Organ and Tissue Authority. Best Practice Guideline for Offering Organ and Tissue Donation in Australia (2017). Available at: https://donatelife.gov.au/resources/clinical-guidelines-and-protocols/best-practice-guideline-offering-organ-and-tissue (Accessed 1 24, 2022).

[15] Cuende N Cuende JI Fajardo J Huet J Alonso M. Effect of Population Aging on the International Organ Donation Rates and the Effectiveness of the Donation Process. Am J Transpl (2007) 7(6):1526–35. 10.1111/j.1600-6143.2007.01792.x 17430401

[16] Weiss J Elmer A Mahíllo B Domínguez-Gil B Avsec D Nanni Costa A Evolution of Deceased Organ Donation Activity versus Efficiency over a 15-year Period: An International Comparison. Transplantation (2018) 102(10):1768–78. 10.1097/tp.0000000000002226 29677069

[17] Goldberg D Karp S Shah MB Dubay D Lynch R. Importance of Incorporating Standardized, Verifiable, Objective Metrics of Organ Procurement Organization Performance into Discussions about Organ Allocation. Am J Transpl (2019) 19(11):2973–8. 10.1111/ajt.15492 31199562

[18] Siminoff LA Gardiner HM Wilson-Genderson M Shafer TJ. How Inaccurate Metrics Hide the True Potential for Organ Donation in the United States. Prog Transpl (2018) 28(1):12–8. 10.1177/1526924818757939 29592635

[19] Rose C Nickerson P Delmonico F Randhawa G Gill J Gill JS. Estimation of Potential Deceased Organ Donors in Canada. Transplantation (2016) 100(7):1558–63. 10.1097/tp.0000000000000947 26479283

[20] Manara AR Murphy PG O’Callaghan G. Donation after Circulatory Death. Br J Anaesth (2012) 108(Suppl. l_1):i108–i121. 10.1093/bja/aer357 22194426

[21] Curtis RMK Manara AR Madden S Brown C Duncalf S Harvey D Validation of the Factors Influencing Family Consent for Organ Donation in the UK. Anaesthesia (2021) 76(12):1625–34. 10.1111/anae.15485 33860929

